# Sunitinib Impairs Oral Mucosal Healing Through Endoplasmic Reticulum Stress-Mediated Keratinocyte Dysfunction

**DOI:** 10.3390/cells15010001

**Published:** 2025-12-19

**Authors:** Jiarui Wang, Lihang Shen, Shuo Chen, Xinyu Wang, Yang He, Yi Zhang

**Affiliations:** Department of Oral and Maxillofacial Surgery, Peking University School of Stomatology, Beijing 100081, China

**Keywords:** medication-related osteonecrosis of the jaw, sunitinib, oral mucosa, endoplasmic reticulum stress, keratinocytes, tight junctions

## Abstract

Medication-related osteonecrosis of the jaw (MRONJ) is a severe adverse event triggered by antiresorptive and/or anti-angiogenic agents, characterized by bone destruction, sequestrum formation, and refractory mucosal defects. Effective mucosal healing can be a critical factor for MRONJ prevention and treatment. While endoplasmic reticulum stress (ER stress) has been implicated in tissue repair, its role in MRONJ-associated mucosal healing impairment remains undefined. This study investigated the effects of the anti-angiogenic drug sunitinib on oral mucosal healing and its underlying mechanisms. A mouse model of palatal mucosal defects was established, RNA-seq, transmission electron microscopy, and morphological analyses were used to assess how sunitinib affects ER function during mucosal repair. Using human oral keratinocytes (HOKs), we further elucidated the subcellular mechanisms through which sunitinib influences cell proliferation, migration, cell cycle progression, tight junctions, and apoptosis via techniques such as qPCR, Western blotting, immunofluorescence, and flow cytometry. Our findings demonstrated that sunitinib might induce significant alterations in the morphology of the ER and mitochondria. Both in vivo and in vitro experiments revealed that sunitinib persistently activates the GRP78 (BIP)/PERK/ATF4/CHOP axis in HOKs. This sustained ER stress can inhibit keratinocytes migration and proliferation, disrupt tight junctions, and trigger the intrinsic mitochondrial apoptotic pathway, ultimately leading to impaired oral mucosal healing and barrier dysfunction. Critically, pharmacological inhibition of ER stress was shown to restore keratinocytes’ function and promote effective mucosal healing. These results indicated that targeting sunitinib-induced persistent ER stress might represent a promising therapeutic strategy to prevent and treat oral mucosal toxicity associated with this drug.

## 1. Introduction

Medication-related osteonecrosis of the jaw (MRONJ) is a severe drug-induced adverse event characterized by progressive destruction of the jawbone and soft tissue toxicity. The incidence of MRONJ after tooth extraction in osteoporosis patients receiving bone-modifying agents is approximately 1%, whereas in cancer patients receiving bone-modifying agents, the incidence can reach 25% to 28% [1,2]. Drugs associated with an increased MRONJ risk include antiresorptive agents (bisphosphonates, denosumab) and anti-angiogenic agents, such as anti-vascular endothelial growth factors (VEGFs) agents, tyrosine kinase inhibitors (TKIs), and corticosteroids. Combination therapy with antiresorptive and anti-angiogenic drugs is increasingly used in cancer and osteoporosis treatment, potentially enhancing clinical benefits [3,4]. However, MRONJ case reports associated with this regimen are increasing, suggesting elevated risks of jawbone necrosis [5]. For example, sunitinib combined with bisphosphonates exacerbates jawbone necrosis and reduces post-debridement wound healing rates in MRONJ [6,7]. Our previous research demonstrated that optimal oral mucosal healing is essential for MRONJ treatment and prevention [8,9,10]. Combination therapies involving anti-angiogenic agents tend to exacerbate necrotic gingival ulcers and soft tissue defects [6,11], suggesting that these agents potentially act as pathogenic drivers of impaired oral mucosal wound healing in MRONJ.

Sunitinib, a multi-targeted tyrosine kinase inhibitor, is a standard first-line therapeutic agent for metastatic renal cell carcinoma, offering convenient oral administration, high specificity, demonstrated clinical efficacy, and favorable tolerability [12]. Sunitinib exerts its anti-tumor activity primarily through inhibition of VEGFRs/platelet-derived growth factor receptors (PDGFRs). It has also demonstrated therapeutic potential in gastrointestinal stromal tumors and pancreatic neuroendocrine tumors [13,14]. However, it may cause off-target effects, i.e., drug-induced toxicities, including cardiovascular adverse events, gastrointestinal reactions, and cutaneous toxicities such as hand–foot syndrome and rashes [15]. Notably, clinical evidence demonstrates that sunitinib may induce oral mucositis, ulcerative lesions, and delayed healing processes [16]. These mucosal adverse events currently represent a significant clinical challenge in sunitinib-treated patients, with their underlying pathophysiological mechanisms not yet fully elucidated.

The endoplasmic reticulum (ER) is a complex and dynamic organelle that is critical for maintaining protein, lipid, and calcium homeostasis. Approximately one-third of the human proteome, including membrane receptors and secreted proteins, is biosynthesized in the ER, where proteins acquire their functional folded conformations [17]. Disruption of ER homeostasis, caused by proteotoxic overload, calcium imbalance, exacerbated oxidative stress, or exogenous insults, elicits ER stress and initiates the unfolded protein response (UPR) signaling cascade [18]. The UPR mainly includes three major signaling pathways: IRE1α/XBP1s, PERK/ATF4/CHOP, and ATF6 [19]. The UPR detects protein-folding perturbations in the ER, regulates gene expression, and adjusts ER function to restore proteostasis, thereby maintaining ER homeostasis and regulating cellular physiology [20]. The adaptive UPR restores ER homeostasis under moderate stress, while the maladaptive UPR executes cell death during irremediable ER stress [21]. Emerging evidence indicates that ER-mediated signaling is a key regulator of epithelial tissue repair. During wound healing, intensive protein synthesis and degradation impose a substantial burden on the ER, ultimately leading to ER stress. When persistent ER stress exceeds the ER’s folding potential, the maladaptive UPR pathway is activated, thereby interrupting tissue regeneration [22]. For example, ultraviolet radiation B inhibits skin healing by upregulating ER stress activity in keratinocytes [23]. Similarly, significantly enhanced ER stress signaling is closely associated with ulcerative colitis pathogenesis [24]. Furthermore, the chemotherapeutic agent 5-fluorouracil upregulates ER stress levels in the oral mucosal epithelium, inducing mucosal inflammation and apoptosis [25]. Therefore, targeting ER stress-related signaling pathways may offer novel therapeutic approaches for impaired oral mucosal barrier function or healing deficiencies.

In this study, we investigated the effects of sunitinib on oral mucosal healing in mice and its underlying pathological mechanisms. Using human oral keratinocytes (HOKs), we further elucidated the role of ER stress-mediated cellular dysfunction. Additionally, we evaluated the therapeutic potential of the ER stress inhibitor 4-phenylbutyric acid (4-PBA) in mitigating sunitinib-induced oral soft tissue toxicity and promoting mucosal regeneration.

## 2. Materials and Methods

### 2.1. Animal Experiments

All animal procedures were approved by Peking University School of Stomatology Institutional Animal Care and Use Committee (PKUSSIACUC, BDKQ-202504140548) and conducted in accordance with national guidelines for laboratory animal welfare and experimental protocols. Eight-week-old female C57BL/6JNifdc mice were purchased from Beijing Vital River Laboratory Animal Technology Co., Ltd. (Beijing, China) All mice were housed under specific pathogen-free (SPF) conditions at Peking University School of Stomatology, with environmental parameters maintained at 22 ± 1 °C, 50 ± 5% relative humidity, and 12 h light/dark cycles. Animals received autoclaved food and water ad libitum, with a maximum of six mice per cage.

Mice were randomly divided into three groups: control group, sunitinib group, and sunitinib + 4-PBA treatment group (hereafter referred to as treated group). Three days prior to palatal mucosal defect creation, the sunitinib and treated groups received 30 mg/kg sunitinib (S7781, Selleck, Shanghai, China) via oral gavage every other day. The dosage was determined based on clinical applications, converted for mouse use through the body surface area-based method, and reference to animal model studies of sunitinib as an anticancer agent [26,27,28]. Sunitinib was dissolved in a vehicle containing 5% DMSO (ST038, Beyotime, Shanghai, China), 40% PEG300 (Y268724, Beyotime, China), 5% Tween 80 (T8360, Solarbio, Beijing, China), and 50% PBS (G4202, Servicebio, Wuhan, China) (The formulation method is referenced from https://www.selleck.cn/products/sunitinib.html, accessed on 10 December 2025). The control group received equivalent volumes of the sunitinib-free vehicle. On day 4, mice were anesthetized with 1% pentobarbital sodium, and a standardized mucosal defect (2.5 mm × 1.5 mm) was created on the right palate extending from the anterior first molar to posterior third molar. Mice received meticulous hemostasis during and after the surgery, followed by a soft, nutrient-rich diet for the first three postoperative days. Following modeling, sunitinib was administered by oral gavage at a dose of 30 mg/kg every other day. The treated group received the ER stress inhibitor 4-PBA (S4125, Selleck, China; dissolved in a solution of 40% PEG300 + 5% Tween 80 + 50% PBS), at a dose of 150 mg/kg via intraperitoneal injection twice weekly. After two weeks of modeling and treatment, mice were euthanized by cervical dislocation. Schematic diagrams of the experimental timeline is provided in the Figure S1A,B. Mice were checked daily for their health status. Those showing severe symptoms (e.g., severe lethargy, inability to eat/drink, slow movement) were euthanized based on animal ethical requirements.

### 2.2. Transcriptome Sequencing Analysis

Appropriate palatal mucosal tissues from mice were collected into pre-labeled grinding tubes. Sequencing work was commissioned to BGI Genomics (Shenzhen, China), and the general steps are described below. Add TRIzol lysis buffer, centrifuge, and then use chloroform, isopropanol, and 75% ethanol to extract RNA. Subsequently, mRNA was enriched with oligo (dT) magnetic beads, reverse-transcribed into cDNA, and amplified by PCR. PCR products were heat-denatured into single-stranded DNA, then circularized using bridge primers to generate single-stranded circular DNA libraries. The constructed library was quality-checked, and after passing the inspection, it was sequenced. Raw sequencing data were filtered using SOAPnuke (v2.2.1). Subsequent analysis, visualization, and data mining employed the Dr. Tom multi-omics platform (https://biosys.bgi.com). Cluster heatmaps are generated based on differential gene expression levels, and differential genes are subjected to Kyoto Encyclopedia of Genes and Genomes (KEGG), Gene Ontology (GO) enrichment analysis, and Gene Set Enrichment Analysis (GSEA).

### 2.3. Wound Healing Assessment

After euthanasia of the mice, the palatal mucosal defect was photographed using a stereomicroscope (AxioZoom.V16, CARL ZEISS, Jena, Germany), and the area of the palatal mucosal defect was measured and analyzed using ImageJ 1.54g.

### 2.4. Morphological Analysis

Mouse palatal tissues were fixed in 4% paraformaldehyde (P0099, Beyotime, China), decalcified with 10% EDTA, routinely dehydrated, and paraffin-embedded. Four μm paraffin sections were prepared and stained with hematoxylin and eosin (H&E, C0105M, Beyotime, China). Masson’s trichrome staining was performed using the kit (C0189S, Beyotime, China) according to the manufacturer’s instructions. The collagen content (%) was calculated as the ratio of the area of blue-stained collagen fibers to the total area of the mucosal lamina propria using Image-Pro Plus 6.0 software.

For immunohistochemical staining (IHC), after deparaffinization and rehydration, antigen retrieval was conducted using EDTA buffer (pH = 9.0, ZSGB-BIO, Beijing, China). Sections were blocked and incubated with primary antibodies at 4 °C overnight (antibody information is provided in the Supplementary Materials Table S1). The next day, endogenous peroxidase was blocked, followed by incubation with the secondary antibody (PV9001, PV9002, ZSGB-BIO, China). Then, stained with DAB reagent (ZLI-9018, ZSGB-BIO, China). The average optical density (AOD) for IHC staining was measured using Image-Pro Plus 6.0 software and subjected to statistical analysis.

We performed tartrate-resistant acid phosphatase (TRAP) staining using a TRAP stain kit (294-67001, FUJIFILM Wako, Osaka, Japan). The procedure was conducted according to the manufacturer’s instructions. Briefly, the staining solution was prepared by mixing the tartrate solution, acid phosphatase substrate solution, and acid phosphatase substrate solution B. Then, cell nuclei were counterstained with the provided nuclear stain reagent. To quantify osteoclast activity, the ratio of TRAP-positive area to bone area on the model side was calculated using Image-Pro Plus 6.0 software.

### 2.5. Transmission Electron Microscopy (TEM)

Fresh mouse palatal mucosa were collected in 1.5 mL centrifuge tubes and fixed overnight at 4 °C in electron microscope fixative (P1127, Solarbio, China) in the dark. The samples were fixed with 1% osmium tetroxide solution (18456, Ted Pella Inc., Redding, CA, USA), dehydrated with graded ethanol, osmotized, embedded, and sectioned into 70 µm ultrathin sections using an ultramicrotome. The sections were stained with uranyl acetate (1261209, SPI, West Chester, PA, USA) staining, washed with distilled water, stained with lead citrate, and observed under a transmission electron microscope (Hitachi HT-7800, Hitachi, Tokyo, Japan) at 80 kV, with images collected and analyzed.

### 2.6. Cell Culture and Processing

Human oral keratinocytes (HOKs), an immortalized cell line procured from Huatuo Biotechnology, Beijing, China (HTX2966), were cultured in DMEM medium (C11995500BT, Gibco, Grand Island, NY, USA) containing 10% fetal bovine serum (12103C, Sigma, St. Louis, MO, USA) and 1% (*v*/*v*) antibiotic-antimycotic (15240-062, Gibco, USA) at 37 °C in a 5% CO_2_ humidified incubator. The cells were divided into three groups: DMSO group, sunitinib group, and sunitinib + 4-PBA group.

### 2.7. Cell Proliferation Assay

HOK cells were seeded into a 96-well plate at 2000 cells/well. Cells were incubated with 10% CCK-8 solution (AQ308, Beijing Aoqing Biotechnology, Beijing, China) for 2 h in the dark. The absorbance of each well was measured at 450 nm using a microplate reader, and the cell proliferation curve from day 0 to day 5 was calculated and plotted.

### 2.8. BrdU Incorporation Assay

When the confluence reached approximately 70–80%, cells were incubated with 10 μM BrdU (19-160, Sigma-Aldrich, St. Louis, MO, USA). After a 12 h incubation, cells were fixed with 4% PFA, treated with 2 M HCl and sodium borate neutralization, permeabilized with 0.1% Triton X-100 (T8200, Solarbio, China), blocked, and incubated with anti-BrdU primary antibody (A20304, Abclonal, Wuhan, China) and secondary antibody. Six random fields per sample were imaged using fluorescence microscopy. BrdU-positive cells were quantified with ImageJ 1.54g.

### 2.9. Cell Migration Assay

HOK cells were seeded into 6-well plates. A reference line was scribed on the well bottom using a marker pen. Three linear scratches were created perpendicular to the reference line using a sterile 200 μL pipette tip. Subsequently, cells were pre-treated with 1 μg/mL mitomycin C (Selleck, S8146, China) for 1 h to inhibit cell proliferation. Wells were gently washed with PBS to remove detached cells; the cells were returned to culture. Images of the wounds were captured at 24 and 36 h post-wounding, and migration rates were analyzed using ImageJ.

### 2.10. Quantitative Real-Time PCR (qPCR)

Mouse palatal mucosa tissue was homogenized in TRIzol reagent (15596018, Invitrogen, Carlsbad, CA, USA), using a high-throughput homogenizer (Qiagen, Germantown, MD, USA). Total RNA was extracted using the AG RNA Isolation Kit (AG21024, Accurate Biology, Changsha, China). cDNA was synthesized using the AG Reverse Transcription Kit according to the manufacturer’s instructions. Then, the cDNA was mixed with SYBR Green (RK21203, Abclonal, China), MiliQ water, and primers to prepare the qPCR reaction mixture. qPCR was performed using the Archimed system (ROCGENE, Shanghai, China). Primer sequences are provided in Supplementary Materials Table S2.

### 2.11. Western Blotting (WB)

Total cellular proteins were extracted using RIPA buffer (HX1862-1, Huaxingbio, Beijing, China) supplemented with protease inhibitor (HX1863, Huaxingbio, China) and phosphatase inhibitor (HX1864, Huaxingbio, China). A BCA kit (23227, Thermo Scientific, Grand Island, NY, USA) was used to quantify the protein concentration and prepare the loading buffer. Proteins were separated on 10% SDS-PAGE gels and transferred to 0.2 μm PVDF membranes (ISEQ00010, Millipore, Burlington, MA, USA). Membranes were blocked with 5% non-fat milk (D8340, Solarbio, China) for 2 h at room temperature, then incubated with primary antibodies overnight at 4 °C. The next day, membranes were incubated with HRP-conjugated secondary antibodies for 1 h at room temperature (antibody details are provided in Supplementary Materials Table S1). To detect different antigens or phosphorylated/non-phosphorylated protein forms on the same blot membrane, Western Blot Stripping Buffer (HX18672, Huaxingbio, China) was used to remove the bound primary/secondary antibody complexes. Proteins were detected using enhanced chemiluminescence reagents (P10300, NCM Biotech, Suzhou, China). Band intensity values were quantified with ImageJ, and target protein signals were normalized using GAPDH as an internal control.

### 2.12. ZO-1 Immunofluorescence Staining

HOK cells were seeded onto cell culture slides. After drug treatment, the slides were fixed with ice-cold methanol at −20 °C for 20 min, blocked with 10% goat serum (C0265, Beyotime, China) for 1 h at room temperature, and incubated overnight at 4 °C with ZO-1 primary antibody (21773-1-AP, Proteintech, Wuhan, China).The next day, the secondary antibody (ab96899, Abcam, Waltham, MA, USA)was incubated at room temperature for 1 h. Nuclei were counterstained with DAPI (C0065, Solarbio, China). Images were acquired using an Olympus FV3000 confocal microscope (Olympus Corporation, Tokyo, Japan).

### 2.13. Flow Cytometric Analysis of Apoptosis

Apoptosis was assessed using the APC Annexin V/PI Kit (640932, Biolegend, San Diego, CA, USA). After drug treatment, cell supernatant and cells were collected, centrifuged, and resuspended in 100 μL Annexin V Binding Buffer. Then, 5 μL Annexin V and 10 μL PI were added, and the mixture was incubated at room temperature in the dark for 15 min. Samples were analyzed immediately and quantification using FlowJo 10.8.1. The gating strategy for flow cytometry analysis is detailed in Figure S4A,B.

### 2.14. Cell Cycle Analysis

After digestion and PBS resuspension, cell pellets were fixed overnight at 4 °C with 70% ethanol. PI staining working solution was prepared according to the instructions of the Cell Cycle and Apoptosis Analysis Kit (C1052, Beyotime, China). Fixed cells were stained with PI solution at 37 °C for 30 min in the dark, analyzed by flow cytometry, and ModfitLT 5.0 was used to analyze cell cycle results.

### 2.15. Reactive Oxygen Species (ROS) Level Detection

Cells were collected and resuspended in 1 mL PBS, and then incubated with 1 μL DCFH-DA (S0034S, Beyotime, China) at 37 °C for 30 min in the dark. After centrifugation, the cells were resuspended in 1 mL PBS and analyzed by flow cytometry.

### 2.16. ATP Detection

According to the ATP Assay Kit instructions (S0026, Beyotime, China), cell samples were lysed and processed with all procedures performed on ice. After centrifugation, supernatants were collected. Standard solution and ATP detection working solution were prepared, samples and standard solution were loaded onto opaque white 96-well plates, and relative light unit (RLU) values were measured using a luminometer (0119001319, PerkinElmer, Waltham, MA, USA). The ATP content in each well was calculated based on the standard curve.

### 2.17. JC-1 Staining

Working solution and staining solution were prepared according to the JC-1 staining kit (C2006, Beyotime, China) instructions. Briefly, HOK cells were treated with JC-1 working solution for 20 min at 37 °C. Then, cells were washed twice with JC-1 buffer. Six random fields per sample were imaged by fluorescence microscopy, and red/green fluorescence ratios were quantified using ImageJ.

### 2.18. Statistical Analysis

All statistical analyses and graphing were conducted using GraphPad Prism 10.1.2. “n” represents the number of mice or the number of independent experimental replicates in vitro. Normality was assessed with the Shapiro–Wilk test, and variance homogeneity was verified using the Brown–Forsythe test. Two groups of samples that met the criteria for normal distribution and variance homogeneity were analyzed using an unpaired *t*-test, while multiple groups were analyzed using one-way analysis of variance (ANOVA). When data were not normally distributed or had unequal variances, two groups were analyzed using an unpaired Welch’s *t*-test, and multiple groups were analyzed using the Kruskal–Wallis test followed by Dunn’s post hoc test. Data were presented as mean ± standard deviation (SD) of the mean. *p* < 0.05 was considered statistically significant. * *p* < 0.05; ** *p* < 0.01; *** *p* < 0.001; **** *p* < 0.0001; ns not significant.

## 3. Results

### 3.1. Sunitinib Impairs Palatal Mucosal Healing and Upregulates Inflammatory Response

**Figure 1:**
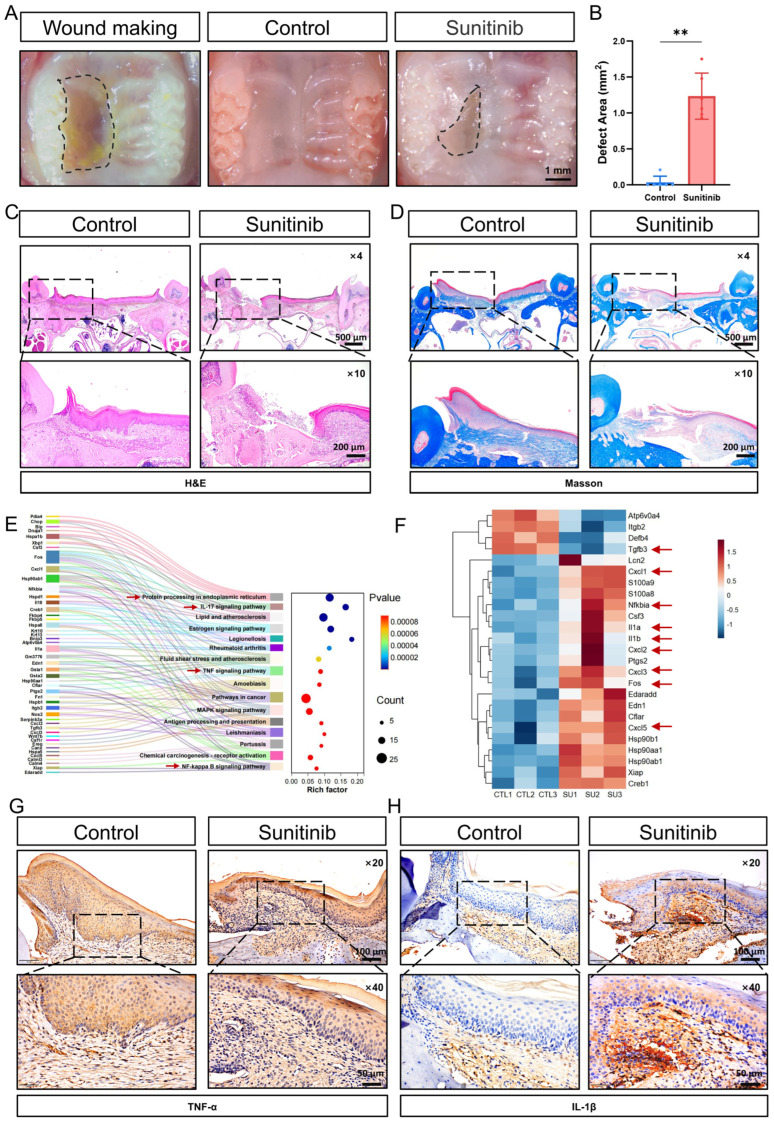
(**A**) Macrograph of mouse palatal mucosal healing at 2 weeks. The dashed boxes outline the wound area; (**B**) measurement of palatal mucosal defect dimensions. *n* = 6 per group. ** *p* < 0.01; (**C**) H&E staining of palatal mucosa; (**D**) Masson’s trichrome staining of palatal mucosa; (**E**) Sankey diagram of DEGs in the palatal mucosa of mice in the control group (CTL1-3) and the sunitinib group (SU1-3). The red arrows highlight the signaling pathways involved in ER and inflammation; (**F**) heatmap of inflammation factors and chemokines DEGs filtered by condition: (FDR *q*-value < 0.05, |log2FC| > 0.5); (**G**) TNFα IHC staining in the palatal mucosa of mouse; (**H**) IL-1β IHC staining in the palatal mucosa of mouse.

To investigate the effects of oral sunitinib administration on mucosal healing, we established a mouse model of unilateral palatal mucosal defect. We found that sunitinib significantly inhibited palatal mucosal healing after 2 weeks of drug administration (Figure 1A,B). H&E staining revealed a complete absence of epithelial and lamina propria structures in non-healed wounds, featuring direct bone exposure and inflammatory cells on the surface (Figure 1C). The adjacent mucosal epithelial papillae were absent, with discontinuous basal cell layers, disorganized granular and spinous cell layers, and unclear stratification, indicating obvious keratinization abnormalities. The surrounding lamina propria displayed extensive fibrous hyperplasia with inflammatory infiltration. Masson’s trichrome staining showed that, compared with the control group, the lamina propria in the sunitinib group exhibited sparse and fragmented, blue-stained collagen fibers (Figure 1D and Figure S2A). Although no distinct sequestrum was formed, the exposed bone area lacking soft tissue coverage exhibited a loss of osteocytes within a limited number of lacunae.

We conducted mRNA sequencing studies on the palatal mucosa of mice in the control group and the sunitinib group. Compared with the control group, the sunitinib group identified a total of 302 differentially expressed genes (DEGs), including 134 upregulated genes and 168 downregulated genes (Figure S1C) (FDR *q*-value < 0.05, |log2FC| > 0.5). The Sankey diagram demonstrated that the differentially expressed genes are primarily enriched in pathways related to protein synthesis and processing, inflammation (NF-κB, TNF-α, etc.), and cell proliferation and differentiation (Figure 1E). The heatmap of inflammation factors and chemokine-related genes showed that pro-inflammatory factors such as *Il1a*, *Il1b*, *Csf3* and *Fos*, as well as chemokines such as *Cxcl2*, *Cxcl3*, and *Cxcl5* were significantly upregulated in the sunitinib group (Figure 1F). IHC staining indicated that inflammatory factors TNFα and IL-1β were markedly increased in the sunitinib group (Figure 1G,H and Figure S2B,C). These results suggest that sunitinib may cause impaired oral mucosal healing and exacerbate the local inflammatory response.

### 3.2. Sunitinib Induces Endoplasmic Reticulum Stress in Oral Mucosa

**Figure 2:**
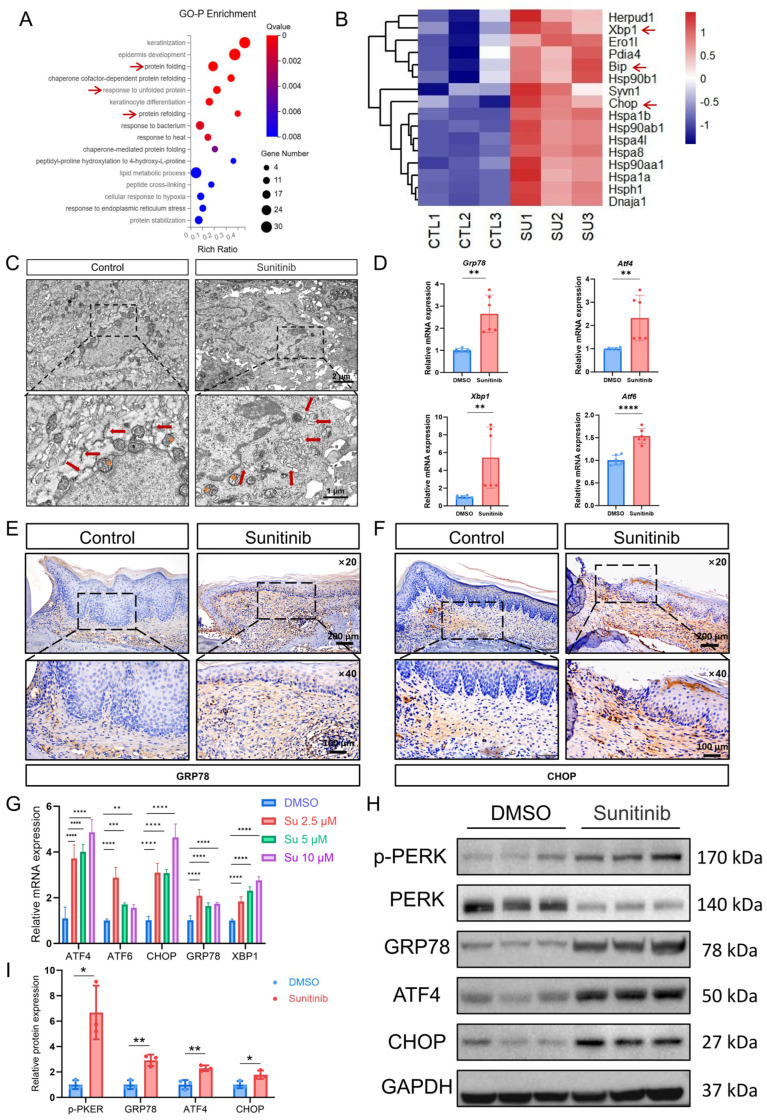
(**A**) Differential gene GO-Biological Process enrichment analysis bubble plot. The red arrows indicate the biological process related to protein synthesis and processing; (**B**) heatmap of endoplasmic reticulum processing and synthesis DEGs filtered by condition (FDR *q*-value < 0.01, |log2FC| > 0.5). The red arrows indicate genes associated with ER stress; (**C**) TEM of the palatal mucosa in control and sunitinib groups showing changes in the endoplasmic reticulum and mitochondria (red arrows indicate the endoplasmic reticulum, orange stars represent mitochondria); (**D**) relative expression levels of ER stress-related genes in mouse palatal mucosa; (**E**) IHC staining of GRP78 in mouse palatal mucosa; (**F**) IHC staining of CHOP in mouse palatal mucosa; (**G**) relative expression levels of ER stress-related genes in HOK cells treated with sunitinib; (**H**) WB analysis of ER stress-related proteins in sunitinib-treated HOK cells. *n * =  3 per group; (**I**) statistical results of WB analysis. * *p* < 0.05; ** *p* < 0.01; *** *p* < 0.001; **** *p* < 0.0001.

We performed GO-Biological Process enrichment analysis. The results showed that protein processing and quality control related pathways showed highly significant enrichment in the sunitinib group (FDR *q*-value < 0.01) (Figure 2A). The heatmap of genes in the ER processing and synthesis pathway showed that UPR-related genes *Grp78*, *Chop*, and *Xbp1* were significantly upregulated in the sunitinib group, suggesting that the sunitinib group exhibited an ER stress response in the palatal mucosa (Figure 2B). The TEM of the sunitinib group mice revealed palatal mucosal keratinocytes with characteristic ER stress morphology: marked ER dilation, swelling, and fragmentation. Concurrently, there were evident signs of mitochondrial damage, including mitochondrial swelling, cristae rupture, and reduced matrix density, indicating impaired mitochondrial structure and function (Figure 2C and Figure S1F). qPCR also showed upregulation of ER stress markers *Grp78*, *Atf4*, *Xbp1*, and *Atf6* in palatal mucosa tissue (Figure 2D). IHC further validated these findings, showing that both proteins, CHOP and GRP78 (BIP), stained strongly in the epithelial cell layer of the drug-treated tissues, in contrast to the weak signals of the controls (Figure 2E,F and Figure S2D,E). These results suggest that sunitinib induces ER stress in oral mucosal keratinocytes, thereby affecting their function during the healing process. Oral mucosal keratinocytes play important roles in the repair of oral mucosal injuries, including acting as a physical barrier, regulating chemical immunity, and maintaining the homeostasis of the mucosal microenvironment [29]. We used HOK cells to further investigate the potential mechanisms of sunitinib. HOK cells were treated with sunitinib (0, 2.5, 5, or 10 μM) for 24 h. qPCR results revealed dose-dependent upregulation of ER stress markers, including *GRP78*, *ATF4*, *ChOP*, *XBP1*, and *ATF6* (Figure 2G), indicating that sunitinib initiates the unfolded protein response as a consequence of ER stress. The upregulation of *GRP78*, *ATF4*, and *CHOP* genes was most significant at a sunitinib concentration of 10 μM, which was consequently used for subsequent experiments. WB results indicated that after 36 h of treatment with 10 μM sunitinib, the expression levels of GRP78/PERK/ATF4/CHOP proteins, which are involved in energy metabolism, oxidative stress regulation, and apoptosis in the UPR, were significantly increased (Figure 2H,I). Collectively, sunitinib over activates ER stress signals, triggering maladaptive UPR via GRP78/PERK/ATF4/CHOP signaling that mediates mucosal healing impairment. To define functional consequences, we further assessed sunitinib’s impact on HOK cells’ proliferation and apoptosis.

### 3.3. Sustained ER Stress Overload Contributes to HOKs Cellular Dysfunction

**Figure 3:**
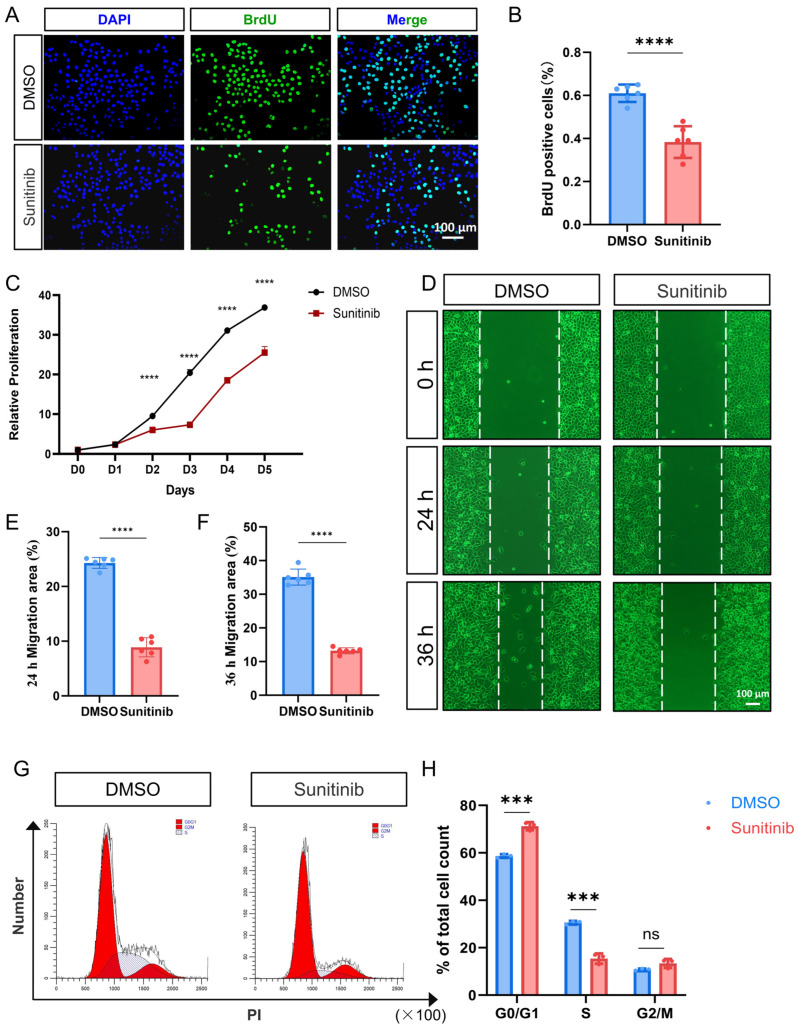
(**A**) BrdU immunofluorescence assay to assess HOK cells proliferation capacity; (**B**) statistical analysis of BrdU immunofluorescence; (**C**) CCK-8 assay to assess HOKs proliferation capacity; (**D**) cell scratch assays to assess HOKs migration capacity; (**E**,**F**) quantification of wound closure ratio; (**G**) flow cytometry analysis of cell cycle results; (**H**) statistical chart of (**G**). *** *p* < 0.001; **** *p* < 0.0001; ns not significant.

Building on evidence that sunitinib hyperactivates ER stress in HOKs, we assessed its functional consequences. We found that sunitinib significantly inhibited the proliferation capacity of HOK cells via CCK-8 assay and BrdU incorporation assay (Figure 3A–C). Cell scratch assays showed that cell migration rates were significantly reduced at 24 h and 36 h post sunitinib treatment compared to the control group (Figure 3D–F). These findings indicate sunitinib compromises keratinocyte recruitment to wound sites, impairing mucosal healing. Cell cycle analysis revealed that sunitinib treatment induced G0/G1 phase arrest in HOK cells, accompanied by a marked reduction in the S-phase population (Figure 3G,H). This result was consistent with the BrdU incorporation assay results.

### 3.4. Sunitinib Affects Tight Junctions Between Cells, Regulates Mitochondrial Function and Apoptosis

**Figure 4:**
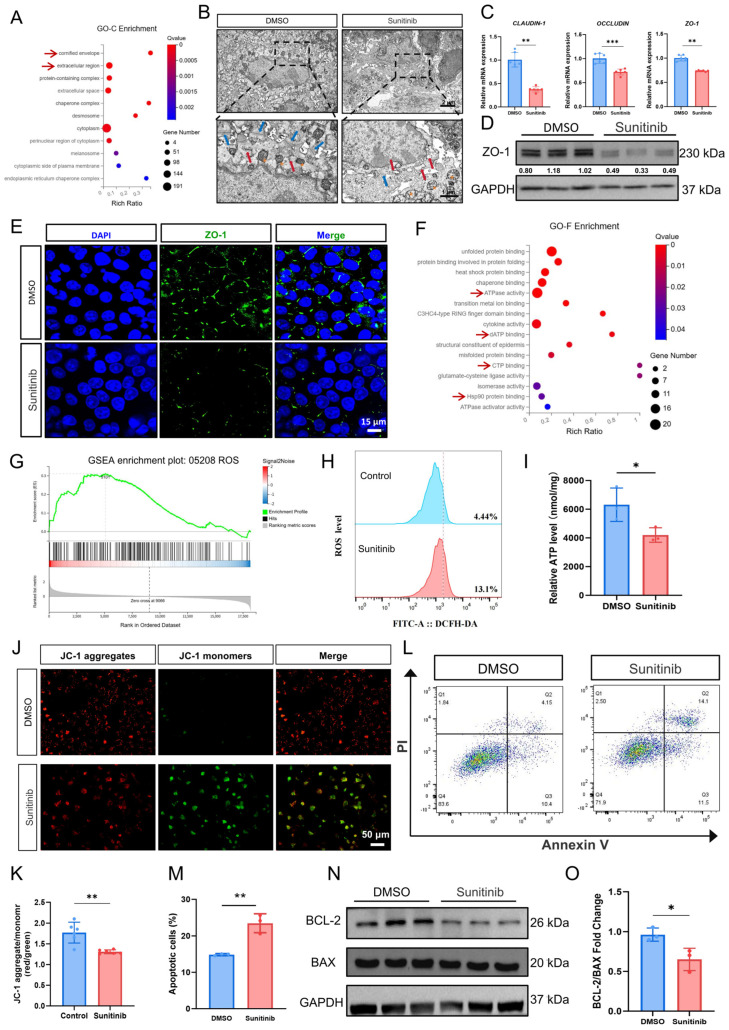
(**A**) Differential gene GO-Cellular Component (GO-C) enrichment analysis bubble plot. The red arrows indicate the Cellular Components related to intercellular connections; (**B**) TEM of mouse palatal mucosa (blue arrows highlight tight junctions, red arrows indicate the endoplasmic reticulum, orange stars represent mitochondria); (**C**) qPCR analysis of tight junction-related gene expression in HOKs; (**D**) WB analysis of ZO-1 protein expression. *n*  =  3 per group; (**E**) immunofluorescence staining of ZO-1 in HOKs; (**F**) bubble plot of GO Function (GO-F) enrichment analysis of DEGs. The red arrows indicate molecular functions related to mitochondrial function; (**G**) GSEA enrichment analysis plot of ROS gene set; (**H**) flow cytometry to detect changes in cellular ROS levels; (**I**) detection of cellular ATP level; (**J**) JC-1 assay to detect changes in mitochondrial membrane potential; (**K**) statistical analysis of JC-1 results; (**L**) flow cytometry analysis of cell apoptosis level in HOKs; (**M**) statistical assessment of HOKs apoptosis levels; (**N**) WB analysis of BCL-2 and BAX protein expression. *n*  =  3 per group; (**O**) statistical results of (**N**). * *p* < 0.05; ** *p* < 0.01; *** *p* < 0.001.

GO-Cellular Component analysis indicated that differentially expressed genes exhibited significant enrichment in epithelial intercellular junction complexes (Figure 4A). Additionally, TEM results revealed significantly widened intercellular spaces between adjacent keratinocytes in the sunitinib-treated palatal mucosa (Figure 4B and Figure S1F), demonstrating a loose distribution pattern indicative of impaired tight junction (TJ) integrity and function. In vitro, qPCR analysis demonstrated sunitinib downregulated the expression levels of tight junction-related genes *Zo-1*, *Occludin*, and *Claudin-1* (Figure 4C). WB analysis further validated that sunitinib significantly downregulated the expression level of ZO-1 (Figure 4D and Figure S1D). To further clarify the changes in ZO-1, immunofluorescence revealed continuous linear ZO-1 localization at cell borders in control HOK cells, forming an intact tight junction network. In contrast, sunitinib-treated cells displayed disrupted ZO-1 distribution with fragmented signals and punctate patterns (Figure 4E).

Flow cytometry analysis and RNAseq results revealed that sunitinib treatment significantly increased ROS levels and apoptosis level within HOKs (Figure 4F–H,L,M). Additionally, ATP detection experiments demonstrated that sunitinib led to a marked reduction in intracellular ATP levels (Figure 4I), suggesting impaired mitochondrial function. Given the activation of the ER stress marker pathway GRP78/PERK/ATF4/CHOP, combined with observed ROS accumulation and ATP depletion, we propose that sunitinib-induced ER stress may promote apoptosis through triggering mitochondrial dysfunction. We detected proteins related to the mitochondrial-mediated intrinsic apoptosis pathway. WB results showed that the ratio of anti-apoptotic protein BCL-2 to pro-apoptotic protein BAX was significantly decreased in the sunitinib group compared to the control group (Figure 4N,O), suggesting enhanced mitochondrial apoptotic susceptibility. Additionally, mitochondrial membrane potential (ΔΨm) was assessed using JC-1 staining. In healthy mitochondria, JC-1 forms red-fluorescent aggregates within the matrix. During a decrease or loss of membrane potential, JC-1 transitions from the polymer form to the monomer form, producing green fluorescence. In our study, after treatment with sunitinib, HOKs exhibited a significant increase in green fluorescence intensity and a decrease in red fluorescence intensity, indicating ΔΨm depolarization (Figure 4J,K).

### 3.5. Inhibition of Excessive ER Stress Signals Improves Cell Dysfunction Caused by Sunitinib

**Figure 5:**
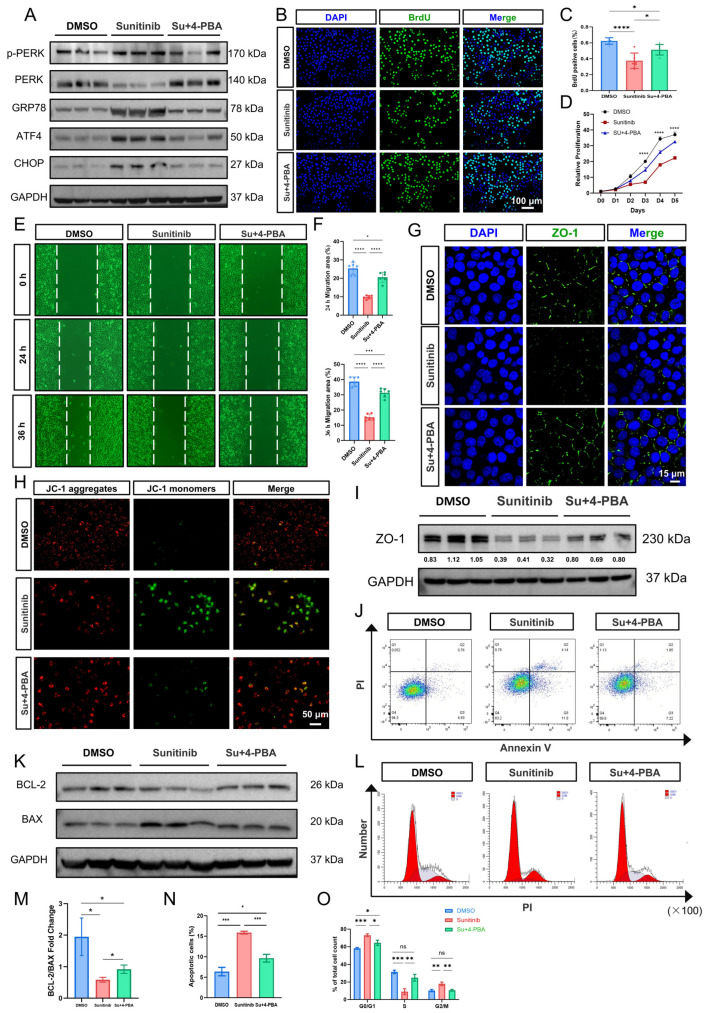
(**A**) WB results of ER stress protein expression levels after 4-PBA treatment; (**B**) BrdU immunofluorescence assay to assess HOK cells proliferation capacity; (**C**) statistical analysis of BrdU immunofluorescence; (**D**) CCK-8 assay to assess HOK cells proliferation capacity; (**E**) scratch assay to detect the migration ability of HOK cells; (**F**) statistical assessment of (**E**), scale bar: 100 μm; (**G**) immunofluorescence staining to visualize ZO-1 expression patterns; (**H**) JC-1 assay to detect changes in mitochondrial membrane potential after 4-PBA treatment; (**I**) WB assay to detect ZO-1 protein expression levels after 4-PBA treatment. *n*  =  3 per group; (**J**) flow cytometry analysis of apoptosis levels; (**K**) WB assay to detect BCL-2 and BAX protein expression levels after 4-PBA treatment. *n*  =  3 per group; (**L**) flow cytometry analysis of cell cycle results; (**M**) statistical results of (**K**); (**N**) statistical assessment of (**J**); (**O**) statistical charts of (**L**). * *p* < 0.05; ** *p* < 0.01; *** *p* < 0.001; **** *p* < 0.0001; ns not significant.

To determine whether overactive ER stress mediates sunitinib-induced HOKs dysfunction and mitochondrial impairment, we applied the ER stress inhibitor 4-PBA in vitro. The chemical compound 4-PBA is an effective chemical chaperone that inhibits ER stress by restoring endoplasmic reticulum protein folding capacity [13]. WB analysis demonstrated that 4-PBA significantly reduced sunitinib-induced ER stress markers GRP78, p-PERK, ATF4, and CHOP (Figure 5A and Figure S2F). ER stress inhibition also significantly restored HOKs proliferation and migration capacity (Figure 5B–F) [30]. Notably, 4-PBA restored both the ZO-1 expression level and the continuous linear localization pattern of ZO-1 (Figure 5G,I and Figure S1E). JC-1 analysis, Annexin V/PI flow cytometry, and WB showed that 4-PBA reduced the proportion of apoptotic cells while inhibiting the expression of apoptosis-related proteins (Figure 5H,J,K,M,N and Figure S2G). Additionally, ER stress inhibition attenuated sunitinib-induced cell cycle arrest, showing a restoration of S phase (Figure 5L,O). These results were also consistent with an increase in the number of BrdU-positive cells. These findings collectively demonstrate that ER stress inhibition effectively rescues sunitinib-induced multiparameter impairment in HOKs.

### 3.6. Inhibition of ER Stress Signals Restores the Sunitinib-Impaired Oral Mucosal Healing in Mice

**Figure 6:**
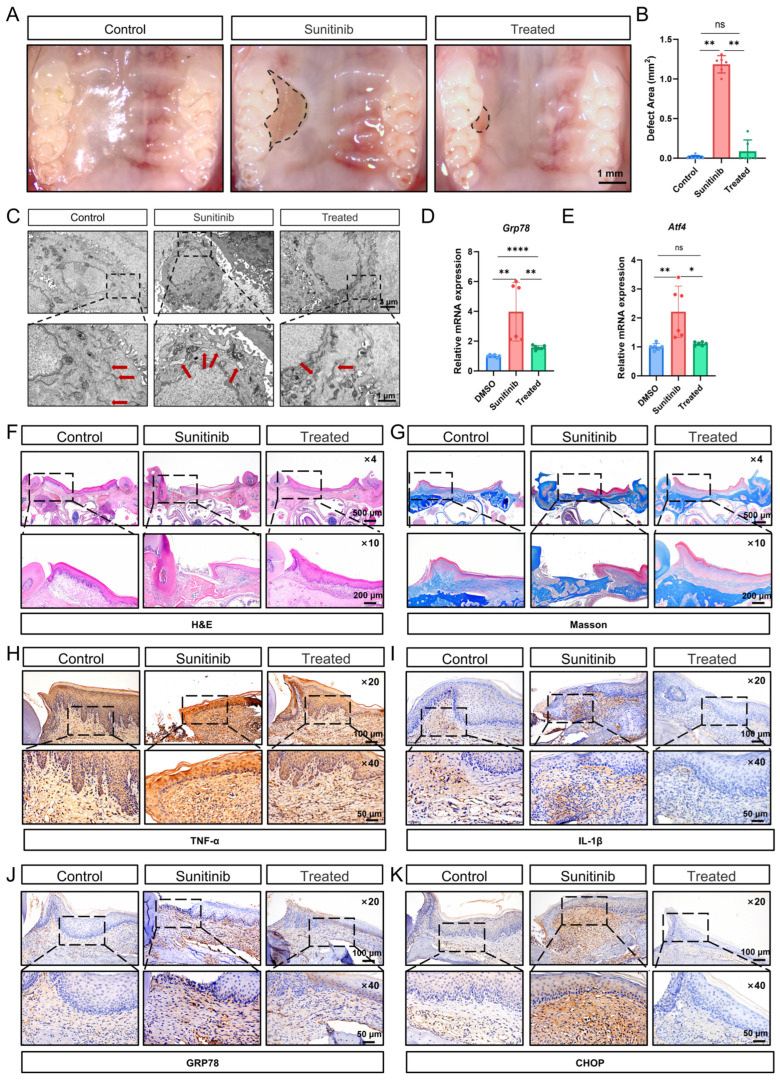
(**A**) Macroscopic of palatal mucosal healing 2 weeks post-wounding. The dashed boxes delineate the unhealed wound area; (**B**) measurement of palatal mucosal defect dimensions. *n* = 6 per group; (**C**) TEM of palatal mucosa (red arrows indicate endoplasmic reticulum); (**D**,**E**) qPCR analysis of ER stress-related genes expression in palatal mucosa; (**F**) H&E staining of palatal mucosa; (**G**) Masson’s trichrome staining of palatal mucosa; (**H**–**K**) IHC staining for TNFα, IL-1β, GRP78, and CHOP in palatal mucosa. * *p* < 0.05; ** *p* < 0.01; **** *p* < 0.0001; ns not significant.

To further validate that sunitinib mediates oral mucosal healing impairment via ER stress hyperactivation, we intraperitoneally administered 4-PBA (twice weekly) to the sunitinib-group mice. After 2 weeks of treatment, 4-PBA significantly alleviated sunitinib-induced mucosal healing impairment, improving wound closure rates (Figure 6A,B). TEM analysis of 4-PBA-treated mice demonstrated that mucosal keratinocytes exhibited flattened ER cisternae with narrowed lumens and ordered arrays. Mitochondria displayed reduced swelling, distinct cristae layers, and uniform matrix electron density (Figure 6C). Notably, 4-PBA treatment significantly suppressed the expression of Grp78 and Atf4 in mouse palatal mucosa (Figure 6D,E). H&E staining demonstrated continuous, intact epithelial stratification on hard palates after 4-PBA treatment (Figure 6F). Masson’s trichrome staining showed thicker, regularly aligned collagen bundles in the treated group compared to the sunitinib group, indicating enhanced mucosal regeneration (Figure 6G and Figure S3A). IHC confirmed reduced inflammatory infiltration and decreased expression of ER stress-related markers in the treated group (Figure 6H–K and Figure S3B–E). Collectively, ER stress inhibition restores sunitinib-induced mucosal healing impairment.

## 4. Discussion

VEGFRs, PDGFRs, and stem cell factor receptor (KIT) are tyrosine kinases that play pivotal roles in regulating tumor proliferation and angiogenesis. As an orally administered multi-targeted tyrosine kinase receptor inhibitor, sunitinib exerts anti-angiogenic and anti-tumor activities in various malignancies by blocking these receptors [31]. Clinically, sunitinib demonstrates significant efficacy, especially combined with immune checkpoint inhibitors, extending median progression-free survival in multiple cancers [32,33]. However, as the clinical use of sunitinib has expanded, reports of its associated adverse effects have progressively increased. Current research has predominantly focused on sunitinib’s resistance mechanisms [32], induction of hypertension [34], and cardiovascular toxicity [35]. In contrast, systematic investigations into its mucosal barrier-related adverse effects, like particularly stomatitis and gastrointestinal mucosal injury, have received limited attention. Indeed, research on sunitinib-associated MRONJ remains limited to case reports and retrospective studies. According to the 2022 American Association of Oral and Maxillofacial Surgeons(AAOMS)’s position paper on MRONJ update, MRONJ likely arises from the combined effects of multiple medications, the underlying disease itself, and the patient’s immunocompromised status. Nevertheless, sunitinib is recognized as a potential risk factor with limited supporting evidence and is listed as a medication requiring vigilance. Further controlled prospective studies remain necessary to quantify its associated risk [36]. The impetus for our investigation into anti-angiogenic agents originated from clinical observations of an increasing number of cases involving combination drug therapies. During the preliminary phase of the research, we evaluated the effects of bevacizumab, sunitinib, and sorafenib on mucosal wound healing in mice. The results demonstrated that the sunitinib group exhibited the most significant impairment in mucosal healing (Figure S2H). In our study, we demonstrate that sunitinib induces mucosal healing impairment, potentially through persistent ER stress and barrier dysfunction of keratinocytes.

During ER stress, misfolded proteins accumulate within the ER lumen. Molecular chaperone GRP78 dissociates from ER transmembrane stress sensors (PERK, IRE1α, ATF6) to bind misfolded proteins, activating the UPR [37], which is a crucial process for tissue repair. Physiological UPR activation promotes tissue regeneration through transient suppression of protein synthesis and upregulation of ER chaperones, thereby enhancing protein folding capacity [38]. In contrast, irreversible ER stress that overwhelms its adaptive response triggers UPR-mediated apoptosis, ultimately suppressing tissue regeneration [22]. Phosphorylated PERK activates ATF4, which transcriptionally upregulates pro-apoptotic factors such as CHOP, thereby initiating apoptosis. In our study, TEM revealed marked ER dilation and swelling in sunitinib-treated palatal mucosa, confirming hyperactive ER stress in keratinocytes. In vivo and in vitro studies demonstrated sunitinib induces pathological ER stress, causing UPR hyperactivation predominantly through the GRP78/PERK/ATF4/CHOP axis.

Rapid wound bed re-epithelialization is critical for restoring oral mucosal barrier function and preventing infection, whereas delayed re-epithelialization directly correlates with poor healing outcomes. Keratinocytes migration and proliferation constitute the pivotal cellular events driving re-epithelialization [39,40]. Our study demonstrated that GRP78/PERK/ATF4/CHOP axis activation arrested HOK cells in the G0/G1 phase, inhibiting S-phase entry and suppressing proliferation process. Consistently, Zhou et al. reported PERK/ATF4/CHOP signaling pathway-induced G1-phase arrest [41]. Sunitinib impairs directional cell migration by inhibiting VEGFR/PDGFR signaling, which disrupts classical migration signals and cellular polarity [42,43]. Our study demonstrated that suppression of pathological ER stress rescued the migratory capacity of HOKs, which is essential for oral mucosal epithelial repair.

Tight junctions constitute critical intercellular structures in epithelia, which is essential for maintaining cell polarity, regulating paracellular permeability, and establishing barrier function [44,45]. As the core scaffolding protein of cellular tight junctions, ZO-1 contributes to both mechanotransduction and the regulation of epithelial proliferation and mucosal healing processes [46,47]. Our findings demonstrate that sunitinib significantly suppressed the transcriptional level of *Claudin*, *Occludin*, and *Zo-1*. This increased oral mucosal permeability to inflammatory mediators and pathogens may further disrupt barrier integrity [48,49], potentially explaining the pronounced inflammatory responses observed in our study. Typically, ZO-1 is widely distributed in epithelial cells with rapid cell turnover rates [50]. Kuo et al. [51] employed epithelial-specific ZO-1 knockout mice and found that ZO-1 maintains the healthy progression of the mucosal healing process by upregulating epithelial proliferation and ensuring the successful completion of mitosis. The formation of tight junction belts is cooperatively driven by adhesion receptor-mediated ZO-1 surface condensation and localized actin polymerization [52]. Consistent with Seo’s findings [53], excessive ER stress induces hyperactivation of the PERK/CHOP axis, resulting in the disruption of tight junctions. This effect is partially attributable to F-actin disorganization secondary to Ca^2+^ depletion. We observed sunitinib-induced alterations in ZO-1 expression and localization. Notably, inhibition of chronic ER stress rescued ZO-1 expression patterns and restored proliferation rates. These findings deepen our understanding of the mechanisms underlying TKI-associated oral mucosal wound healing impairment.

Notably, the TEM of mouse palatal mucosa revealed sunitinib-induced mitochondrial swelling and increased mitochondria-associated endoplasmic reticulum membrane (MAM) contacts. Concurrently, GSEA revealed significant enrichment of the mitochondria-associated MAM gene set (Figure S4C), suggesting alterations in mitochondrial-ER tethering structures. MAMs represent dynamic interorganellar junctions between the ER and the outer mitochondrial membrane (OMM), maintaining an approximately 10–30 nm intermembrane distance via protein complexes (such as IP3R1/GRP75/VDAC1). These structures facilitate intercompartmental communication and metabolite exchange while preventing fusion. Dynamic MAMs interactions modulate intracellular Ca^2+^ homeostasis, protein trafficking, metabolic fluxes, apoptosis, and autophagy [54,55]. During mitochondrial dysfunction, impaired ATP synthesis and accumulated ROS ultimately lead to the collapse of the mitochondrial membrane potential and cellular damage [56]. Our study demonstrated that suppressing ER stress rescued sunitinib-induced apoptosis and mitochondrial dysfunction. Consistent with altered ΔΨm, elevated ROS, and an increased BAX/BCL-2 ratio, these data suggest that sunitinib-induced apoptosis might be partially mediated by the mitochondrial pathway. The widespread MAMs phenomenon implies that sunitinib might disrupt Ca^2+^ signaling through aberrant ER-to-mitochondria transmission, thereby inducing mitochondrial damage. However, the precise molecular mechanisms by which ER-mitochondria crosstalk regulates cell survival require further investigation.

Critically, it is important to note that there is no evidence suggesting that sunitinib directly induces the skin or mucosal tissue wound. Our findings merely extend current understanding of the mechanisms underlying sunitinib-induced mucosal toxicity in pre-existing wounds, establishing an ER stress-initiated pathological cascade that modulates cell fate decisions, accompanied by inflammatory responses and resulting in epithelial barrier dysfunction. We also evaluated the therapeutic potential of 4-PBA, which is approved by the Food and Drug Administration for treating urea cycle disorders [57]. The chemical compound 4-PBA not only alleviates global ER stress but also functions as a molecular chaperone to mediate protein folding correction, thereby directly stabilizing proteins. A study demonstrated that 4-PBA could alleviate particulate matter induced inflammatory outcomes in bronchial epithelium [58]. Furthermore, 4-PBA ameliorates allergic rhinitis and restores epithelial barrier function through ER stress alleviation [59]. Critically, this approach targets upstream ER stress rather than downstream inflammatory mediators [59]. This strategy may offer novel therapeutic avenues for managing sunitinib-associated mucosal toxicity.

However, this study has several limitations that should be acknowledged. Oral mucosal wounds typically initiate angiogenesis within days, characterized by increased capillary density near the wound margins to facilitate nutrient delivery and cellular recruitment for tissue repair [29,60], which directly influences tissue repair and re-epithelialization. As a tyrosine kinase inhibitor, sunitinib treatment inevitably inhibits angiogenesis. Investigating the impact on keratinocytes is only one of the reasons for sunitinib-induced healing impairment. We still need to consider the oral mucosa as an integrated microenvironment and comprehensively evaluate the potential adverse effects of sunitinib on immune cells, fibroblasts, vascular endothelial cells, and other components. Secondly, as a calcium reservoir, the ER plays a crucial role in intracellular calcium regulation. Although we have assessed ER stress levels through morphological and protein analyses, future studies should employ calcium fluorescent probes to dynamically track calcium signaling. This approach may further provide a theoretical basis for subsequent research on ER-mitochondria interactions mediated by calcium signaling. Additionally, sunitinib impairs the structural and functional integrity of MAMs in mouse palatal mucosa, but the specific molecular mechanisms underlying MAMs damage still require further investigation.

The AAOMS has recently cautioned clinicians about sunitinib’s off-target effects, with rising reports of MRONJ associated with its use. When prescribing sunitinib, clinicians should be aware of its potential to contribute to MRONJ, particularly in patients with additional risk factors such as tooth extraction or other oral surgical procedures [36]. Our experimental group design was primarily established to simulate the typical clinical scenario at patient presentation. Specifically, early-stage lesions often fail to attract sufficient attention from both patients and clinicians. Consequently, in the presence of pre-existing soft tissue wounds, most patients are likely to continue their medication as prescribed. MRONJ pathogenesis is simultaneously regulated by multiple factors, including oral microbiota and mucosal inflammation and repair, as well as bone metabolism and remodeling. An imbalance in any of these three aspects can act as an initiating factor, driving disease onset and progression. Therefore, it is particularly important to alert both oncologists and dental surgeons to focus on early oral mucosal lesions in these patients. We hope that our findings can, to some extent, serve as an alert and help secure a critical therapeutic window for more patients.

Notably, Vallina et al. [7] systematically reviewed 9 years of sunitinib-associated osteonecrosis-of-the-jaw cases, demonstrating that sunitinib monotherapy significantly inhibits jawbone remodeling and promotes osteonecrosis. However, Li et al. [28] reported no significant disruption of bone metabolic homeostasis in sunitinib monotherapy mouse maxillae. In our study, we utilized COL1A, RUNX2, and OCN as indicators of osteogenic/osteoblastic activity, and TRAP staining as an indicator of osteoclast activity (Figure S3F–K). Combined with H&E and Masson staining results, the findings suggest that sunitinib did not appear to significantly affect overall palatal bone metabolism. However, we observed that in the sunitinib-treated group, a small number of bone lacunae lacked osteocytes specifically at the exposed bone sites without soft tissue coverage (this phenomenon was not observed on the non-surgical contralateral side). Such findings were absent in the fully healed submucosal bone of both the control and treated groups. This is likely attributable to our surgical procedure, which involved subperiosteal flap elevation, leading to compromised soft tissue coverage and disruption of periosteal blood supply, thereby affecting the quality and viability of the bone in these areas. Nonetheless, osteoclast activity and positive osteoblast presence were still detectable in the abnormal bone regions. We hypothesize that this bone is undergoing a remodeling process, making it difficult to determine based on current results whether sunitinib can directly induce osteonecrosis. Given that inflammation and infection are known risk factors for MRONJ, it is possible that a longer observation period and/or more aggressive inflammatory stimulation may be required to determine whether a classic MRONJ sequestrum can form in our study.

## 5. Conclusions

In summary, we demonstrate that sunitinib can induce persistent ER stress in oral mucosal keratinocytes, inhibits proliferation and migration, compromises tight junction integrity, and triggers mitochondrial pathway-dependent apoptosis, ultimately contributing to the impairment of oral mucosal wound healing (Graphical Abstract). The ER stress inhibitor 4-PBA was shown to effectively attenuate these pathological manifestations. Our research could provide a new perspective on the mechanisms of clinical side effects induced by anti-angiogenic drugs, suggesting that targeting severe ER stress might represent a potential therapeutic strategy.

## Data Availability

The datasets generated and/or analyzed during this study are available from the author upon reasonable request.

## Supplementary Materials

The following supporting information can be downloaded at: https://www.mdpi.com/article/10.3390/cells15010001/s1, Figure S1. (A,B) Schematic diagrams of experimental timeline; IP: Intraperitoneal injection; (C) Volcano plot for DEGs; (D) Statistical results of WB analysis of ZO-1 protein expression treated with sunitinib; (E) Statistical results of WB analysis of ZO-1 protein expression after 4-PBA treatment; (F) TEM of mouse palatal mucosa (Blue arrows highlight tight junctions, red arrows indicate endoplasmic reticulum, orange stars represent mitochondria). Figure S2. (A) Quantitative analysis of relative collagen content in the lamina propria of Figure 1D. (B) The AOD value of TNF-α in Figure 1G. (C) The AOD value of IL-1β in Figure 1H. (D) The AOD value of GRP78 in Figure 2E. (E) The AOD value of CHOP in Figure 2F. (F) Statistical results of WB assay of ER stress protein expression levels after 4-PBA treatment. (G) Statistical analysis of JC-1 results after 4-PBA treatment. (H) Mice received bevacizumab through intraperitoneal injection, sunitinib through oral gavage, and sorafenib through oral gavage. Oral mucosal healing was then assessed using stereomicroscopy at the two-week postoperative time point. Figure S3. (A) Quantitative analysis of relative collagen content in the lamina propria of Figure 6G. (B) The AOD value of TNF-α in Figure 6H. (C) The AOD value of IL-1β in Figure 6I. (D) The AOD value of GRP78 in Figure 6J. (E) The AOD value of CHOP in Figure 6K. (F) TRAP staining results. (G) Percentage of TRAP-positive cell area in bone. (H) IHC staining results for COL1A1. (I) Quantitative analysis of COL1A1 positive area in the lamina propria. (J) IHC staining results for RUNX2. (K) IHC staining results for OCN. Figure S4. (A,B) The gating strategy for apoptosis flow cytometry analysis of Figure 4L and Figure 5J; (C) GSEA enrichment analysis plot of MAM gene set. Table S1. Antibodies information. Table S2. qPCR primer information.

## Abbreviations

The following abbreviations are used in this manuscript:

MRONJ	medication-related osteonecrosis of the jaw
ER stress	endoplasmic reticulum stress
DEGs	differentially expressed genes
UPR	unfolded protein response
HOKs	human oral keratinocytes
4-PBA	4-phenylbutyric acid
TKIs	tyrosine kinase inhibitors
KIT	stem cell factor receptor
TJ	Tight junction
VEGFRs	vascular endothelial growth factor receptors
PDGFRs	platelet-derived growth factor receptors
AAOMS	According to the American Association of Oral and Maxillofacial Surgeons
MAM	mitochondria-associated endoplasmic reticulum membrane
KEGG	Kyoto Encyclopedia of Genes and Genomes
GO	Gene Ontology
GSEA	Gene Set Enrichment Analysis
OMM	outer mitochondrial membrane
IP	Intraperitoneal injection

## References

[1] Avishai G., Muchnik D., Masri D., Zlotogorski-Hurvitz A., Chaushu L. (2022). Minimizing MRONJ after Tooth Extraction in Cancer Patients Receiving Bone-Modifying Agents. J. Clin. Med..

[2] Hasegawa T., Hayashida S., Kondo E., Takeda Y., Miyamoto H., Kawaoka Y., Ueda N., Iwata E., Nakahara H., Kobayashi M. (2019). Medication-related osteonecrosis of the jaw after tooth extraction in cancer patients: A multicenter retrospective study. Osteoporos. Int..

[3] Schmouchkovitch A., Remaud M., Simon H., Herry H., Le Toux G., Boisrame S. (2018). Focus: Drug-related osteonecrosis of the jaw. Presse Méd..

[4] Keizman D., Ish-Shalom M., Pili R., Hammers H., Eisenberger M.A., Sinibaldi V., Boursi B., Maimon N., Gottfried M., Hayat H. (2012). Bisphosphonates combined with sunitinib may improve the response rate, progression free survival and overall survival of patients with bone metastases from renal cell carcinoma. Eur. J. Cancer.

[5] Fantasia J.E. (2015). The Role of Antiangiogenic Therapy in the Development of Osteonecrosis of the Jaw. Oral Maxillofac. Surg. Clin. N. Am..

[6] Fusco V., Porta C., Saia G., Paglino C., Bettini G., Scoletta M., Bonacina R., Vescovi P., Merigo E., Lo Re G. (2015). Osteonecrosis of the Jaw in Patients with Metastatic Renal Cell Cancer Treated with Bisphosphonates and Targeted Agents: Results of an Italian Multicenter Study and Review of the Literature. Clin. Genitourin. Cancer.

[7] Vallina C., Ramirez L., Torres J., Casanas E., Hernandez G., Lopez-Pintor R. (2019). Osteonecrosis of the jaws produced by sunitinib: A systematic review. Med. Oral Patol. Oral Cir. Bucal.

[8] Zheng Y., Wang X., He Y., Chen S., He L., Zhang Y. (2024). Exosomes from Adipose-Derived Mesenchymal Stromal Cells Prevent Medication-Related Osteonecrosis of the Jaw by Inhibiting Macrophage M1 Polarization and Pyroptosis. Int. J. Nanomed..

[9] Dong X., He L., Zang X., He Y., An J., Wu B., Liu X., Bi H., Zhang Y., Xiao E. (2021). Adipose-Derived Stem Cells Promote Bone Coupling in Bisphosphonate-Related Osteonecrosis of the Jaw by TGF-beta1. Front. Cell Dev. Biol..

[10] Dong X., He Y., An J., He L., Zheng Y., Wang X., Wang J., Chen S., Zhang Y. (2023). Increased apoptosis of gingival epithelium is associated with impaired autophagic flux in medication-related osteonecrosis of the jaw. Autophagy.

[11] Smidt-Hansen T., Folkmar T.B., Fode K., Agerbaek M., Donskov F. (2013). Combination of zoledronic Acid and targeted therapy is active but may induce osteonecrosis of the jaw in patients with metastatic renal cell carcinoma. J. Oral Maxillofac. Surg..

[12] Rini B.I., Hutson T.E., Figlin R.A., Lechuga M.J., Valota O., Serfass L., Rosbrook B., Motzer R.J. (2018). Sunitinib in Patients with Metastatic Renal Cell Carcinoma: Clinical Outcome According to International Metastatic Renal Cell Carcinoma Database Consortium Risk Group. Clin. Genitourin. Cancer.

[13] Serrano C., Martin-Broto J., Asencio-Pascual J.M., Lopez-Guerrero J.A., Rubio-Casadevall J., Bague S., Garcia-Del-Muro X., Fernandez-Hernandez J.A., Herrero L., Lopez-Pousa A. (2023). 2023 GEIS Guidelines for gastrointestinal stromal tumors. Ther. Adv. Med. Oncol..

[14] Oberg K. (2018). Management of functional neuroendocrine tumors of the pancreas. Gland Surg..

[15] Vignand-Courtin C., Martin C., Le Beller C., Mateus C., Barbault-Foucher S., Rieutord A. (2012). Cutaneous side effects associated with sunitinib: An analysis of 8 cases. Int. J. Clin. Pharm..

[16] Villa A., Lodolo M., Sonis S. (2025). Oral mucosal toxicities in oncology. Expert Opin. Pharmacother..

[17] Wright M.T., Plate L. (2021). Revealing functional insights into ER proteostasis through proteomics and interactomics. Exp. Cell Res..

[18] Tsai Y.C., Weissman A.M. (2010). The Unfolded Protein Response, Degradation from Endoplasmic Reticulum and Cancer. Genes Cancer.

[19] Read A., Schroder M. (2021). The Unfolded Protein Response: An Overview. Biology.

[20] Karagoz G.E., Acosta-Alvear D., Walter P. (2019). The Unfolded Protein Response: Detecting and Responding to Fluctuations in the Protein-Folding Capacity of the Endoplasmic Reticulum. Cold Spring Harb. Perspect. Biol..

[21] Ajoolabady A., Kaplowitz N., Lebeaupin C., Kroemer G., Kaufman R.J., Malhi H., Ren J. (2023). Endoplasmic reticulum stress in liver diseases. Hepatology.

[22] Minjares M., Thepsuwan P., Zhang K., Wang J. (2025). Unfolded protein responses: Dynamic machinery in wound healing. Pharmacol. Ther..

[23] Tai M., Chen J., Chen J., Shen X., Ni J. (2024). Endoplasmic reticulum stress in skin aging induced by UVB. Exp. Dermatol..

[24] Tan Y., Shen S., Li X., Yi P., Fu B., Peng L. (2024). Mogroside V reduced the excessive endoplasmic reticulum stress and mitigated the Ulcerative colitis induced by dextran sulfate sodium in mice. J. Transl. Med..

[25] Cao Y., Wang Y., Zhang L., Hou Y., Shan J., Li M., Chen C., Zhou Y., Shan E., Wang J. (2022). Protective effect of endoplasmic reticulum stress inhibition on 5-fluorouracil-induced oral mucositis. Eur. J. Pharmacol..

[26] Wang H., Chen Y., Yang Y., Song R., Gu S., Cao X., Zhang L., Yang Y., Hou T., Qi X. (2025). MAGI3 enhances sensitivity to sunitinib in renal cell carcinoma by suppressing the MAS/ERK axis and serves as a prognostic marker. Cell Death Dis..

[27] Wang H., Zhang L., Liu H., Yang Y., Lu W., Cao X., Yang X., Qin Q., Song R., Feng D. (2024). PDZK1 confers sensitivity to sunitinib in clear cell renal cell carcinoma by suppressing the PDGFR-beta pathway. Br. J. Cancer.

[28] Li Q., Li Y., Qiao Q., Zhao N., Yang Y., Wang L., Wang Y., Guo C., Guo Y. (2023). Oral administration of Bifidobacterium breve improves anti-angiogenic drugs-derived oral mucosal wound healing impairment via upregulation of interleukin-10. Int. J. Oral Sci..

[29] Groeger S., Meyle J. (2019). Oral Mucosal Epithelial Cells. Front. Immunol..

[30] Jia L., Jingzhen Z., Xinliang Y., Bishao S., Xin L., Ji Z., Zhenqiang F. (2023). 4-PBA inhibits endoplasmic reticulum stress to improve autophagic flux in the treatment of protamine/lipopolysaccharide-induced interstitial cystitis in rats. Sci. Rep..

[31] Faivre S., Demetri G., Sargent W., Raymond E. (2007). Molecular basis for sunitinib efficacy and future clinical development. Nat. Rev. Drug Discov..

[32] Wang Q., Gao S., Shou Y., Jia Y., Wei Z., Liu Y., Shi J., Miao D., Miao Q., Zhao C. (2023). AIM2 promotes renal cell carcinoma progression and sunitinib resistance through FOXO3a-ACSL4 axis-regulated ferroptosis. Int. J. Biol. Sci..

[33] McDermott D.F., Huseni M.A., Atkins M.B., Motzer R.J., Rini B.I., Escudier B., Fong L., Joseph R.W., Pal S.K., Reeves J.A. (2018). Clinical activity and molecular correlates of response to atezolizumab alone or in combination with bevacizumab versus sunitinib in renal cell carcinoma. Nat. Med..

[34] Baek Moller N., Budolfsen C., Grimm D., Kruger M., Infanger M., Wehland M., Magnusson N.E. (2019). Drug-Induced Hypertension Caused by Multikinase Inhibitors (Sorafenib, Sunitinib, Lenvatinib and Axitinib) in Renal Cell Carcinoma Treatment. Int. J. Mol. Sci..

[35] Mahdi A., Wernly B., Pernow J., Zhou Z. (2021). Sunitinib and its effect in the cardiovascular system. Drug Discov. Today.

[36] Ruggiero S.L., Dodson T.B., Aghaloo T., Carlson E.R., Ward B.B., Kademani D. (2022). American Association of Oral and Maxillofacial Surgeons’ Position Paper on Medication-Related Osteonecrosis of the Jaws-2022 Update. J. Oral Maxillofac. Surg..

[37] Zhang T., Li N., Sun C., Jin Y., Sheng X. (2020). MYC and the unfolded protein response in cancer: Synthetic lethal partners in crime?. EMBO Mol. Med..

[38] Vanhoutte D., Schips T.G., Vo A., Grimes K.M., Baldwin T.A., Brody M.J., Accornero F., Sargent M.A., Molkentin J.D. (2021). Thbs1 induces lethal cardiac atrophy through PERK-ATF4 regulated autophagy. Nat. Commun..

[39] Xue M., Jackson C.J. (2015). Extracellular Matrix Reorganization During Wound Healing and Its Impact on Abnormal Scarring. Adv. Wound Care.

[40] Turabelidze A., Guo S., Chung A.Y., Chen L., Dai Y., Marucha P.T., DiPietro L.A. (2014). Intrinsic differences between oral and skin keratinocytes. PLoS ONE.

[41] Zhou D., Yin M., Kang B., Yu X., Zeng H., Chen B., Wang G., Song Y., Liu X., He Q. (2024). CCT020312 exerts anti-prostate cancer effect by inducing G1 cell cycle arrest, apoptosis and autophagy through activation of PERK/eIF2alpha/ATF4/CHOP signaling. Biochem. Pharmacol..

[42] Fan X., Tong Y., Chen Y., Chen Y. (2022). Sunitinib Reduced the Migration of Ectopic Endometrial Cells via p-VEGFR-PI3K-AKT-YBX1-Snail Signaling Pathway. Anal. Cell. Pathol..

[43] Lichner Z., Saleeb R., Butz H., Ding Q., Nofech-Mozes R., Riad S., Farag M., Varkouhi A.K., Dos Santos C.C., Kapus A. (2019). Sunitinib induces early histomolecular changes in a subset of renal cancer cells that contribute to resistance. FASEB. J..

[44] Horowitz A., Chanez-Paredes S.D., Haest X., Turner J.R. (2023). Paracellular permeability and tight junction regulation in gut health and disease. Nat. Rev. Gastroenterol. Hepatol..

[45] Otani T., Furuse M. (2020). Tight Junction Structure and Function Revisited. Trends Cell Biol..

[46] Kuo W., Odenwald M.A., Turner J.R., Zuo L. (2022). Tight junction proteins occludin and ZO-1 as regulators of epithelial proliferation and survival. Ann. N. Y. Acad. Sci..

[47] Vasileva E., Spadaro D., Rouaud F., King J.M., Flinois A., Shah J., Sluysmans S., Mean I., Jond L., Turner J.R. (2022). Cingulin binds to the ZU5 domain of scaffolding protein ZO-1 to promote its extended conformation, stabilization, and tight junction accumulation. J. Biol. Chem..

[48] Van Itallie C.M., Fanning A.S., Bridges A., Anderson J.M. (2009). ZO-1 stabilizes the tight junction solute barrier through coupling to the perijunctional cytoskeleton. Mol. Biol. Cell.

[49] Turner J.R. (2009). Intestinal mucosal barrier function in health and disease. Nat. Rev. Immunol..

[50] Imafuku K., Iwata H., Natsuga K., Okumura M., Kobayashi Y., Kitahata H., Kubo A., Nagayama M., Ujiie H. (2023). Zonula occludens-1 distribution and barrier functions are affected by epithelial proliferation and turnover rates. Cell Prolif..

[51] Kuo W., Zuo L., Odenwald M.A., Madha S., Singh G., Gurniak C.B., Abraham C., Turner J.R. (2021). The Tight Junction Protein ZO-1 Is Dispensable for Barrier Function but Critical for Effective Mucosal Repair. Gastroenterology.

[52] Sun D., Zhao X., Wiegand T., Martin-Lemaitre C., Borianne T., Kleinschmidt L., Grill S.W., Hyman A.A., Weber C., Honigmann A. (2025). Assembly of tight junction belts by ZO1 surface condensation and local actin polymerization. Dev. Cell.

[53] Seo S.H., Kim S., Lee S.E. (2020). ER stress induced by ER calcium depletion and UVB irradiation regulates tight junction barrier integrity in human keratinocytes. J. Dermatol. Sci..

[54] Liu Y., Qiao Y., Pan S., Chen J., Mao Z., Ren K., Yang Y., Feng Q., Liu D., Liu Z. (2023). Broadening horizons: The contribution of mitochondria-associated endoplasmic reticulum membrane (MAM) dysfunction in diabetic kidney disease. Int. J. Biol. Sci..

[55] Missiroli S., Patergnani S., Caroccia N., Pedriali G., Perrone M., Previati M., Wieckowski M.R., Giorgi C. (2018). Mitochondria-associated membranes (MAMs) and inflammation. Cell Death Dis..

[56] Palma F.R., Gantner B.N., Sakiyama M.J., Kayzuka C., Shukla S., Lacchini R., Cunniff B., Bonini M.G. (2024). ROS production by mitochondria: Function or dysfunction?. Oncogene.

[57] Wang Z., Zheng S., Gu Y., Zhou L., Lin B., Liu W. (2020). 4-PBA Enhances Autophagy by Inhibiting Endoplasmic Reticulum Stress in Recombinant Human Beta Nerve Growth Factor-Induced PC12 cells After Mechanical Injury via PI3K/AKT/mTOR Signaling Pathway. World Neurosurg..

[58] Pu L., Yi F., Yu W., Li Y., Tu Y., Xu A., Wang Y. (2023). Endoplasmic reticulum stress mediates environmental particle-induced inflammatory response in bronchial epithelium. J. Immunotoxicol..

[59] Zhang H., Zhu Y., Yang C., Fu L., Huang X. (2025). LncRNA FOXD3-AS1 modulates ER stress and epithelial barrier dysfunction in allergic rhinitis by destabilizing CHOP mRNA. Cell. Signal..

[60] Waasdorp M., Krom B.P., Bikker F.J., van Zuijlen P.P.M., Niessen F.B., Gibbs S. (2021). The Bigger Picture: Why Oral Mucosa Heals Better Than Skin. Biomolecules.

