# Supplementary material for: Sunitinib Impairs Oral Mucosal Healing Through Endoplasmic Reticulum Stress-Mediated Keratinocyte Dysfunction
Source: Cells. 2025 Dec 19;15(1):1. doi: 10.3390/cells15010001 (PMC12784725; doi:10.3390/cells15010001)
Supplement: Supplementary file 1 [file cells-15-00001-s001.zip › cells-4038626-supplementary.pdf]

Supplementary Materials

Figure S1

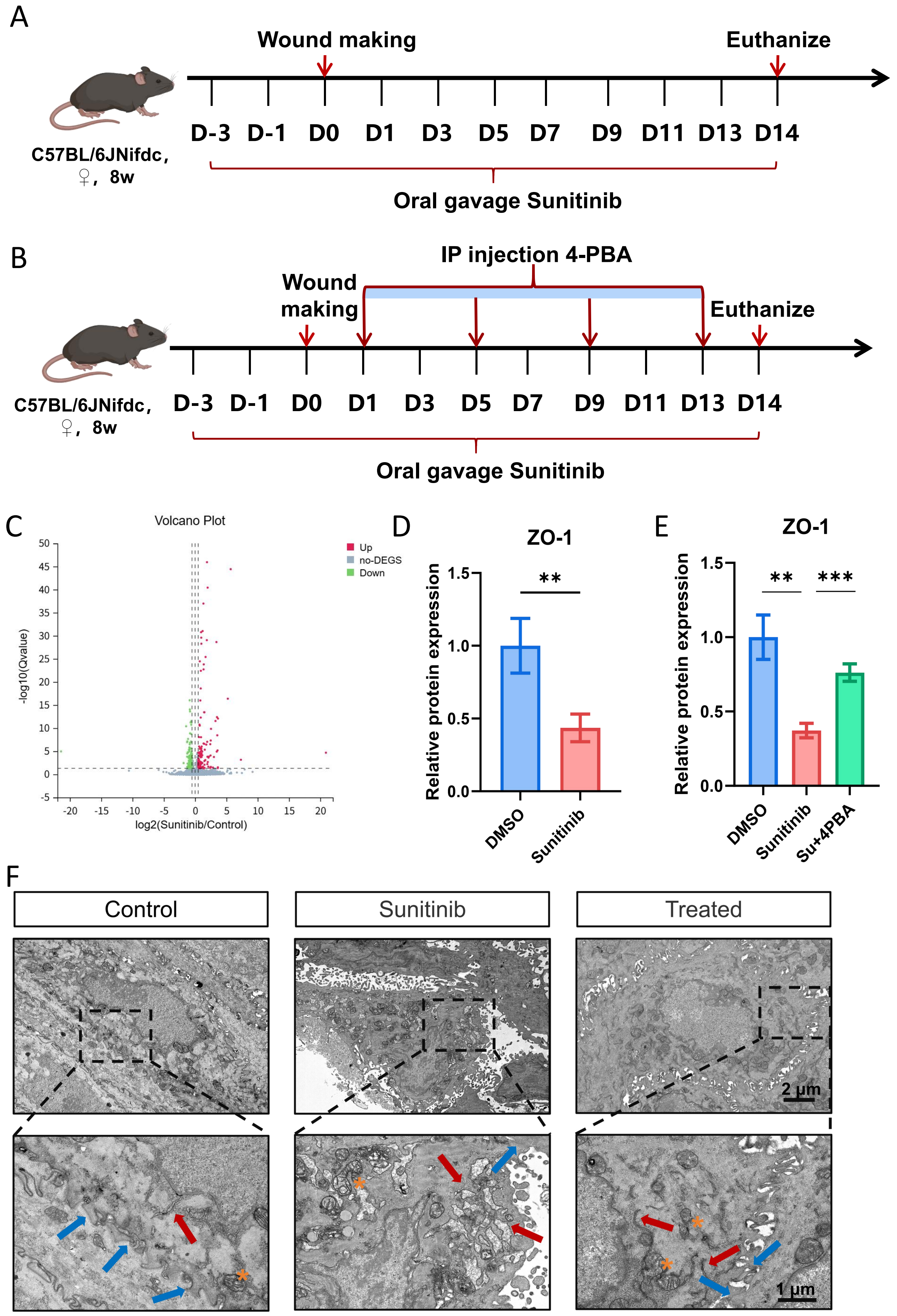

Supplementary Materials

Figure S2

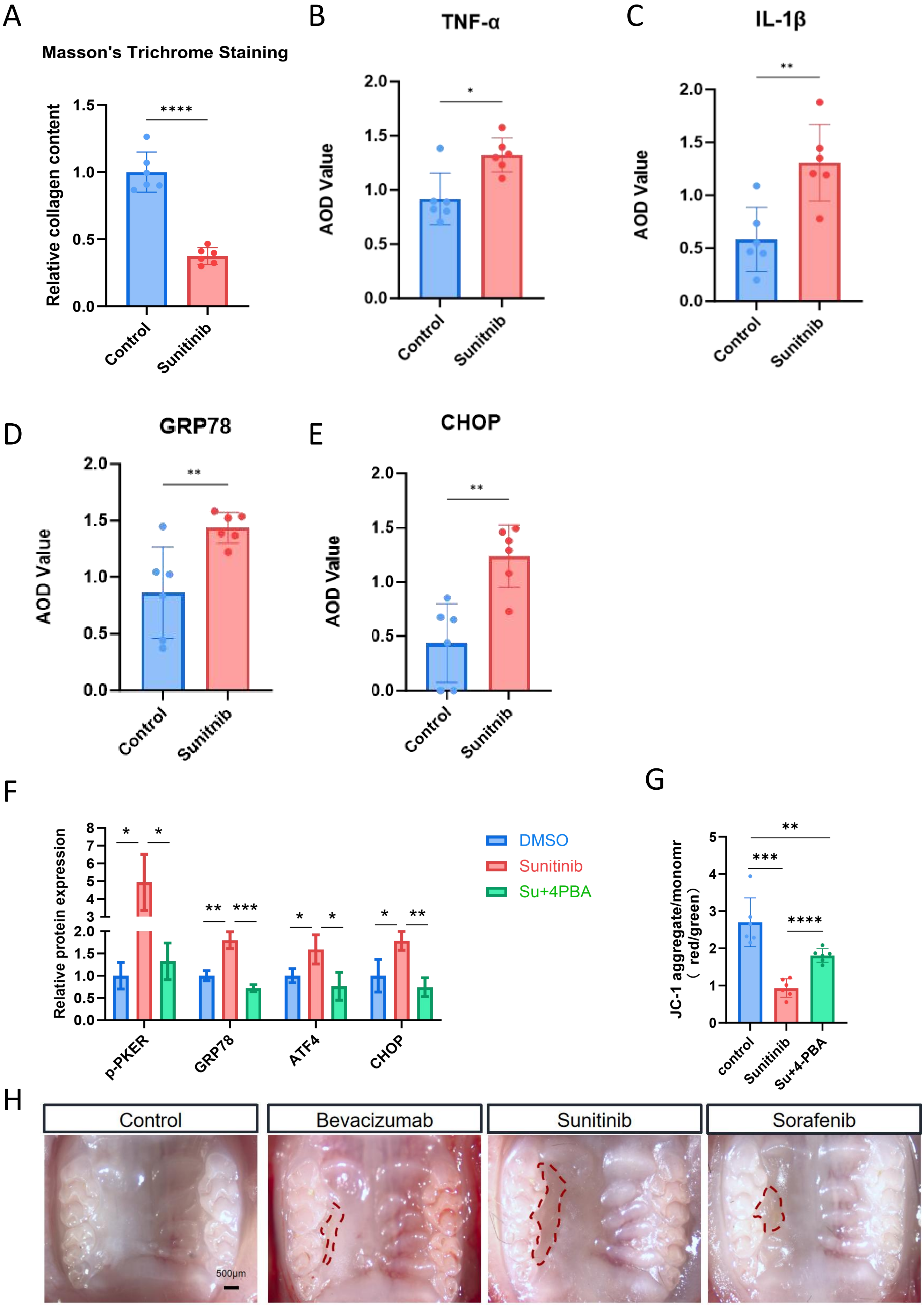

Supplementary Materials

Figure S3

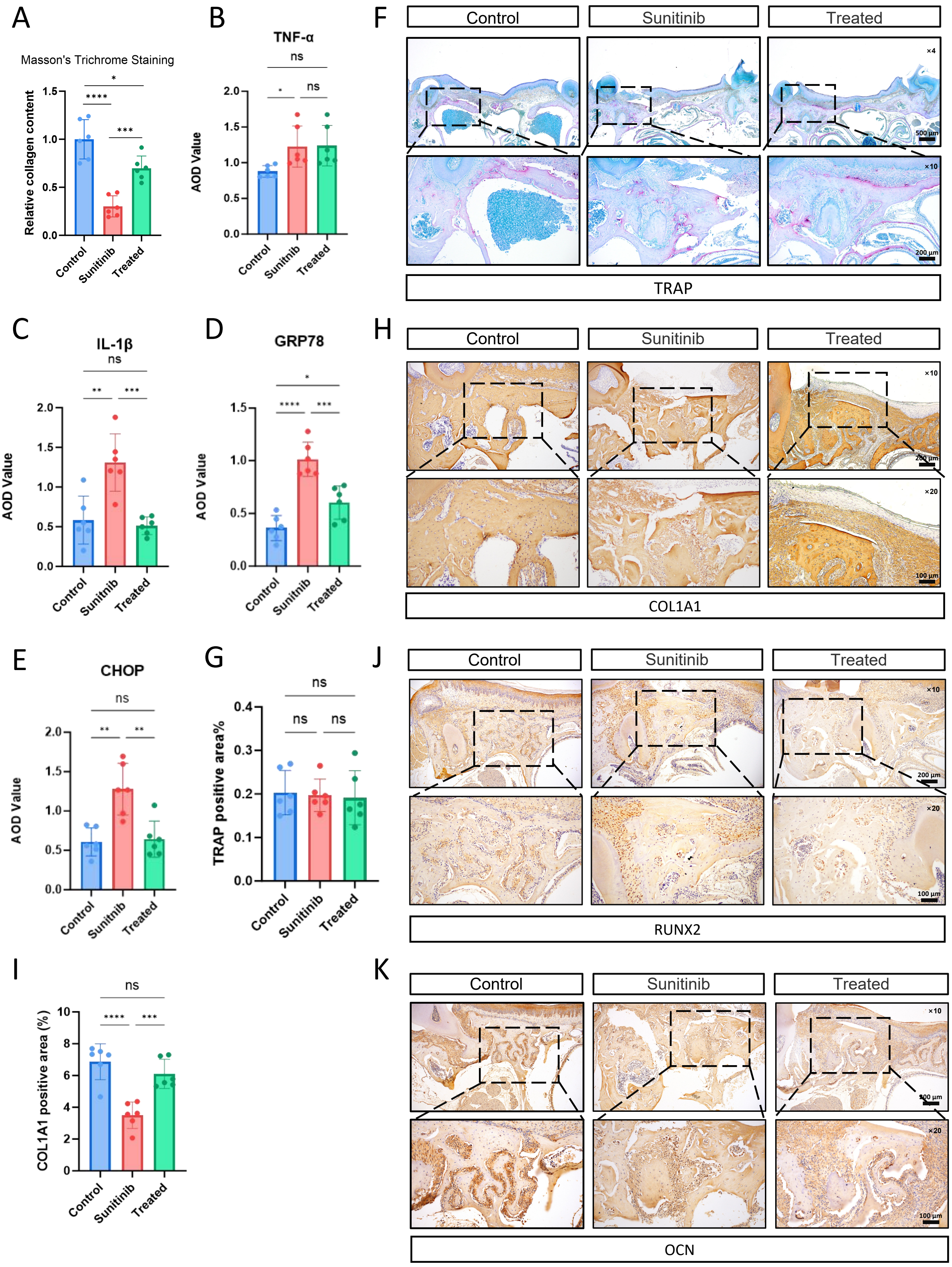

Supplementary Materials

Figure S4

A

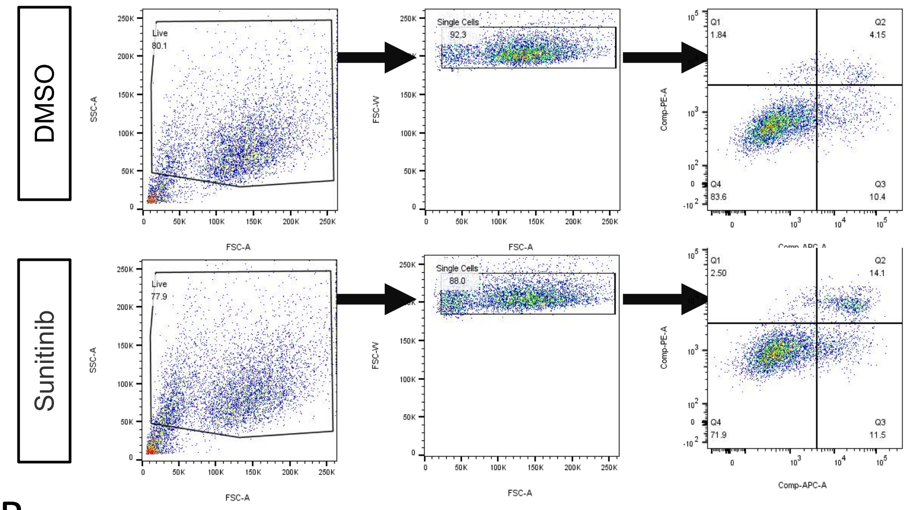

B

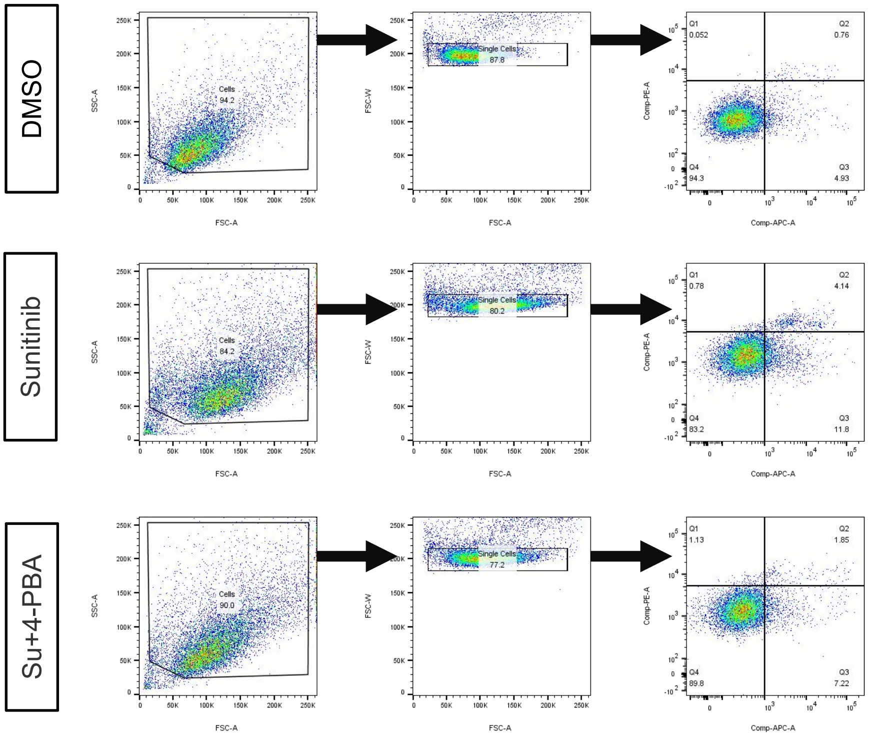

C

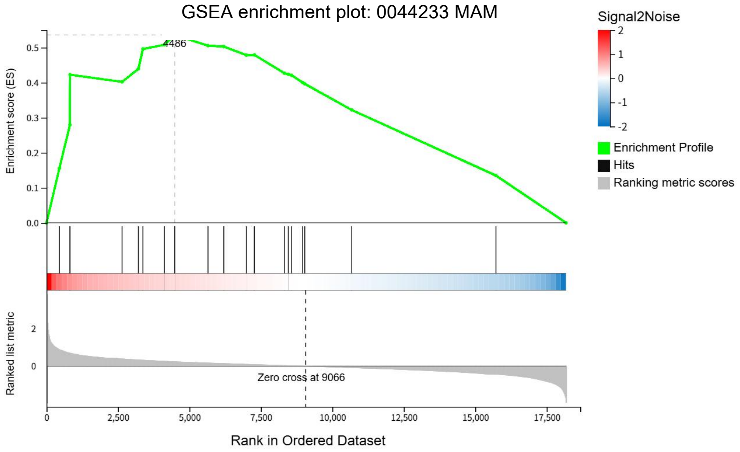

Supplementary Table S1. Antibody Information

Antibody	Brand	Applications and Dilution
BAX	CST 2772S	WB 1:1000
BCL-2	CST 4223S	WB 1:1000
p-PERK	Abclonal AP1501	WB 1:1000
PERK	Selleck F0027	WB 1:1000
ZO-1	Proteintech 21773-1-AP	WB 1:1000<br>IF 1:200
ATF-4	Proteintech 60035-1-IG	WB 1:1000
CHOP	CST 2895	WB 1:1000<br>IHC 1:500
GAPDH	Abclonal A19056	WB 1:100000
GRP78	Abcam ab21685	WB 1:1000<br>IHC 1:500
TNF-α	Abcam ab1793	IHC 1:40
IL-1β	CST 12242	IHC 1:200
COL1A1	CST 72026	IHC 1:200
RUNX2	CST 12556	IHC 1:100
OCN	Abclonal A6205	IHC 1:100

Supplementary Table S2. qPCR Primer Information

Gene	Primer	Sequence (5' → 3')
mouse *Grp78*	Forward	ACTTGGGGACCACCTATTCCT
	Reverse	GTTGCCCTGATCGTTGGCTA
mouse *Chop*	Forward	AAGCCTGGTATGAGGATCTGC
	Reverse	TTCCTGGGGATGAGATATAGGTG
mouse *Atf4*	Forward	CCTGAACAGCGAAGTGTTGG
	Reverse	TGGAGAACCCATGAGGTTTCAA
mouse *Xbp1*	Forward	AGCAGCAAGTGGTGGATTTG
	Reverse	GAGTTTTCTCCCGTAAAAGCTGA
mouse *Atf6*	Forward	TCGCCTTTTAGTCCGGTTCTT
	Reverse	GGCTCCATAGGTCTGACTCC
mouse *Gapdh*	Forward	AGGTCGGTGTGAACGGATTTG
	Reverse	GGGGTCGTTGATGGCAACA
human *CLAUDIN-1*	Forward	AGCTGCAAAATGTACGACTCG
	Reverse	GGAGACCATTAGGGCTC
human *ZO-1*	Forward	CAACATACAGTGACGCTTCACA
	Reverse	CACTATTGACGTTTCCCCACTC
human *OCCLUDIN*	Forward	ACAAGCGGTTTTATCCAGAGTC
	Reverse	GTCATCCACAGGCGAAGTTAAT
human *GRP78*	Forward	CATCACGCCGTCCTATGTCG
	Reverse	CGTCAAAGACCGTGTTCTCG
human *CHOP*	Forward	GGAAACAGAGTGGTCATTCCC
	Reverse	CTGCTTGAGCCGTTCATTCTC
human *ATF4*	Forward	ATGACCGAAATGAGCTTCCTG
	Reverse	GCTGGAGAACCCATGAGGT
human *ATF6*	Forward	TCCTCGGTCAGTGGACTCTTA
	Reverse	CTTGGGCTGAATTGAAGGTTTTG
human *GAPDH*	Forward	GGAGCGAGATCCCTCCAAAAT
	Reverse	GGCTGTTGTCATACTTCTCATGG
human *XBP1*	Forward	CCCTCCAGAACATCTCCCCAT
	Reverse	ACATGACTGGGTCCAAGTTGT
